# Mechanical Properties of Hybrid Fiber-Recycled Concrete and Flexural Performance of Its BFRP-Reinforced Beams

**DOI:** 10.3390/ma19173607

**Published:** 2026-08-25

**Authors:** Buyun Xu, Pan Wu, Jiakun Zhu, Xiaolei Li, Xiaochun Fan

**Affiliations:** 1 School of Civil Engineering and Architecture, Wuhan University of Technology, Wuhan 430070, China; 18627997836whut@sina.com (B.X.); ppwu@foxmail.com (P.W.); 18827357137whut@sina.com (X.L.); fxcfree@126.com (X.F.); 2 School of Mechanical and Electrical Engineering, Hubei Polytechnic Institute, Xiaogan 432000, China; 3 The College of Post and Telecommunication, Wuhan Institute of Technology, Wuhan 430070, China

**Keywords:** RAC, BFRP bar, composite beam, flexural performance

## Abstract

The combined use of recycled aggregate concrete (RAC) and basalt fiber-reinforced polymer (BFRP) bars offers a promising sustainable and corrosion-resistant solution for reinforced concrete structures. However, the inferior quality of recycled aggregates and the relatively low elastic modulus of BFRP bars can compromise the mechanical and flexural performance of RAC members. To address these issues, hybrid fiber-reinforced recycled aggregate concrete (HFRAC) incorporating polyvinyl alcohol (PVA) and steel fibers was developed, and its mechanical and flexural performances were experimentally investigated. The basic mechanical properties of conventional Portland cement concrete (PC), fiber-free RAC, and RAC with hybrid fiber (HF) contents of 0.6%, 0.9%, 1.2%, and 1.5% were first evaluated. A total of nine beams were subsequently tested under four-point bending to investigate the effects of HF content (0–1.5%) and BFRP reinforcement ratio (0.48–1.98%) on flexural behavior. The results showed that an HF content of 1.2% provided the best performance among the investigated fiber contents at both the material and structural levels. At the material level, compared with RAC, 1.2% HF increased the cube compressive strength, axial compressive strength, elastic modulus and splitting tensile strength by 19.44%, 23.08%, 11.39% and 32.55%, respectively. The incorporation of HF effectively mitigated the mechanical deterioration caused by recycled aggregates, allowing HFRAC to achieve comparable or improved basic mechanical properties relative to RAC. At the structural level, compared with the fiber-free RAC beam, the beam with 1.2% HF exhibited increases of 132.51% and 11.92% in cracking and ultimate loads, respectively, and a 47.9% reduction in crack width, while also demonstrating improved flexural performance compared with the PC beam under the investigated conditions. Three failure modes were observed, namely BFRP bar rupture, balanced failure, and concrete crushing, with balanced failure occurring at a reinforcement ratio of approximately 1.0–1.1%. The hybrid fibers effectively refined cracks through a bridging effect, demonstrating superior crack control compared to increasing the reinforcement ratio alone. This study offers valuable insights into improving the performance of RAC and facilitating the wider adoption of BFRP bars in structural applications.

## 1. Introduction

The accelerated pace of urbanization and infrastructure development has led to a considerable increase in construction and demolition waste (CDW), posing growing concerns regarding resource consumption and environmental sustainability [1]. The use of recycled aggregate concrete (RAC), produced by replacing natural aggregates with recycled aggregates derived from demolished concrete, has been widely recognized as an effective approach to promote sustainable construction and reduce the consumption of natural resources [2,3]. Previous studies have demonstrated that the incorporation of recycled aggregates can reduce the environmental impacts associated with concrete production, including carbon emissions and landfill burden [3,4]. However, owing to the presence of adhered mortar, microcracks, and other inherent defects in recycled aggregates, RAC generally exhibits lower strength and stiffness, as well as greater variability, than conventional concrete [5,6,7,8]. These deficiencies can limit the structural application of RAC, particularly in members where crack control and deformation performance are critical.

Meanwhile, fiber-reinforced polymer (FRP) reinforcement has gained increasing attention as a viable substitute for conventional steel bars, especially for structures subjected to aggressive environmental conditions. Among various FRP types, basalt fiber-reinforced polymer (BFRP) bars are characterized by their high tensile capacity, inherent resistance to corrosion, and favorable long-term durability, which makes them well suited for sustainable structural applications in marine and coastal environments [9,10]. In contrast to steel reinforcement, BFRP bars remain linearly elastic throughout the loading process until failure and are characterized by a comparatively low elastic modulus. Consequently, BFRP-reinforced concrete members are generally more susceptible to excessive deflection and wider cracks under service loads [11,12,13].

The combination of RAC and BFRP bars provides a potentially sustainable and corrosion-resistant structural system. However, their respective deficiencies may become coupled when used together. The relatively low stiffness of BFRP reinforcement, combined with the lower strength, stiffness, and greater brittleness of the RAC matrix, can lead to inadequate deformation control and more pronounced cracking in BFRP-reinforced RAC members [14,15]. Previous studies have reported that BFRP-reinforced RAC beams may exhibit larger deflections, wider crack widths, and less favorable failure characteristics than their counterparts incorporating conventional concrete [16,17,18]. Therefore, improving the mechanical and post-cracking properties of RAC is essential for enhancing the serviceability and structural applicability of BFRP-reinforced RAC members.

Fiber reinforcement provides an effective means of improving the mechanical and deformation-related performance of RAC [13]. Through crack bridging and stress transfer across developing cracks, fibers can enhance tensile resistance, suppress crack propagation, improve post-cracking behavior, and promote a more balanced failure response [19,20,21,22]. In particular, hybrid fiber (HF) systems combining fibers with different mechanical properties, geometries, and reinforcing mechanisms can provide complementary effects across different stages of cracking [23,24]. Compared with single-fiber reinforcement, HF systems have demonstrated greater potential for improving strength, toughness, crack control, and post-cracking performance through synergistic fiber-bridging mechanisms [25,26,27,28]. Moreover, previous studies have demonstrated that fiber reinforcement, particularly HF reinforcement, can effectively mitigate the excessive crack widths and deflections of FRP-reinforced concrete beams associated with the relatively low elastic modulus of FRP bars [29,30,31,32,33]. The use of HF may therefore provide an effective approach to simultaneously addressing the inferior mechanical properties of RAC and the serviceability limitations associated with the low stiffness of BFRP reinforcement. A recent study by Htet et al. [25] investigated hybrid fiber-reinforced RAC beams reinforced with BFRP bars or steel–BFRP composite bars and demonstrated the effectiveness of hybrid fibers in improving flexural performance and ductility. However, that study primarily focused on comparing different reinforcement types and evaluating the overall flexural response of FRRAC beams. The effects of hybrid fiber content on the fundamental mechanical properties of RAC and its interaction with BFRP reinforcement ratio in controlling crack development, deformation behavior, and failure modes were not systematically investigated.

Although recent studies have demonstrated the feasibility of HF reinforcement in BFRP-reinforced RAC beams, the mechanisms governing the combined effects of HF characteristics and BFRP reinforcement parameters remain insufficiently understood. In particular, previous investigations have mainly emphasized the improvement in overall flexural capacity and ductility, whereas the influence of HF content on the mechanical properties of RAC and its subsequent contribution to crack control, deformation behavior, and failure mode transition of BFRP-reinforced RAC beams requires further clarification. Moreover, the interaction between HF content and BFRP reinforcement ratio in determining the structural response of these sustainable composite members has not been systematically investigated.

Therefore, this study develops a hybrid fiber-reinforced recycled aggregate concrete (HFRAC) incorporating polyvinyl alcohol (PVA) fibers and steel fibers and systematically investigates the relationship between fiber modification and structural performance of BFRP-reinforced RAC beams. The effects of HF content on the fundamental mechanical properties of RAC are first evaluated. Subsequently, the coupled influences of HF content and BFRP reinforcement ratio on the failure modes, load-deflection response and crack development of BFRP-reinforced HFRAC beams are investigated.

## 2. Experimental Materials and Methods

### 2.1. Materials and Their Characteristics

Ordinary Portland cement (P.O 42.5) produced by Hubei Huaxin Cement Co., Ltd. (Huangshi, China) served as the main binder in this study. Two types of fibers, namely 12-mm-long steel fibers obtained from Hebei Demai Wire Mesh Products Co., Ltd. (Hengshui, China) and high-strength polyvinyl alcohol (PVA) fibers provided by Shanghai Chenqi Chemical Technology Co., Ltd. (Shanghai, China), were employed to modify the mechanical and cracking characteristics of the recycled aggregate concrete (RAC). The former primarily contributed to crack bridging and mechanical enhancement, whereas the latter was introduced to improve tensile capacity and post-cracking behavior. The relevant physical and mechanical characteristics of the steel and PVA fibers are summarized in Table 1.

**Figure 1:**
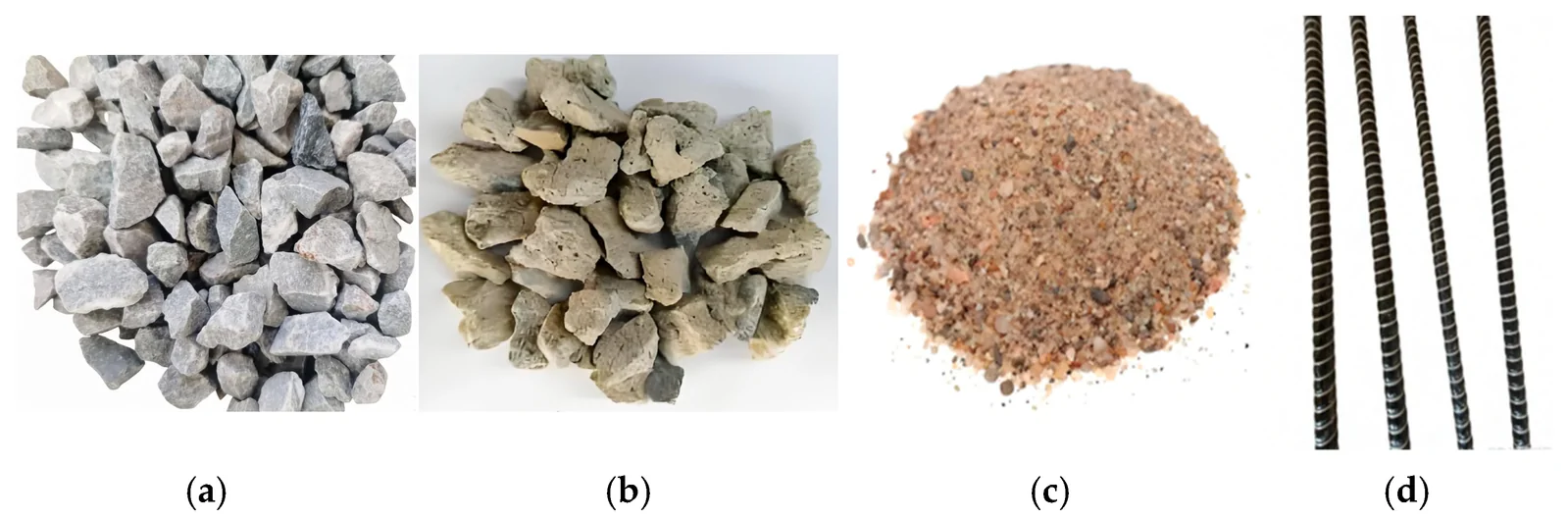
Materials used in this study: (**a**) natural coarse aggregate; (**b**) recycled coarse aggregate; (**c**) river sand; and (**d**) BFRP bars.

**Figure 2:**
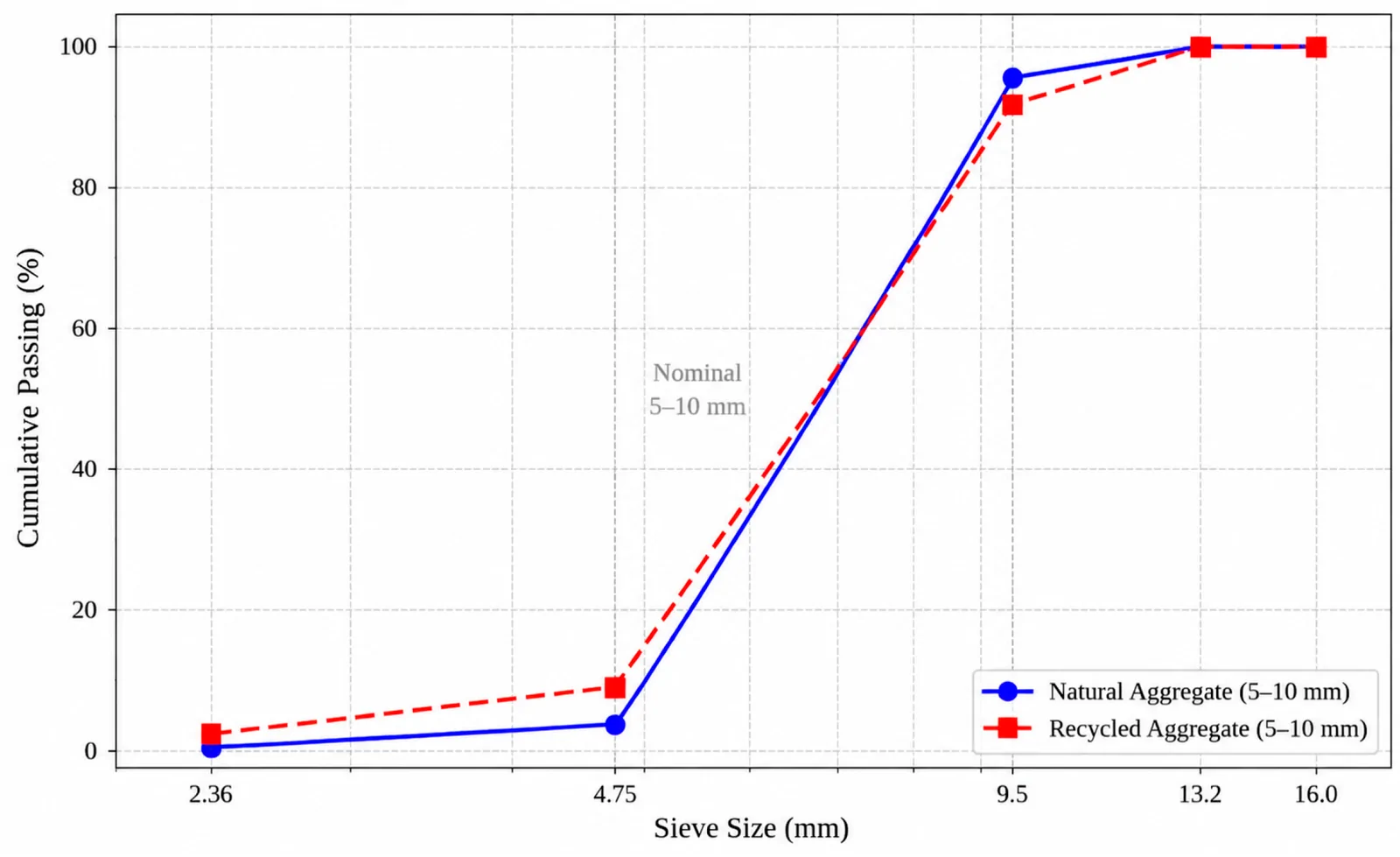
Particle size distribution curves of NCA and RCA.

Recycled coarse aggregate (RCA) was produced from waste concrete collected at a construction site in Hubei Province, China. After manual crushing, the waste concrete was processed by sieving to obtain RCA particles in the 5–10 mm size range. Natural coarse aggregate (NCA) and river sand were adopted as the coarse and fine aggregates, respectively, with the latter having a fineness modulus of 2.4. BFRP bars manufactured by Jiangsu Green Material Valley New Material Technology Development Co., Ltd. (Nanjing, China) were employed for both the longitudinal reinforcement and stirrups of the test beams. Longitudinal reinforcement consisted of BFRP bars with nominal diameters of 8, 12, 14, and 16 mm, whereas 8-mm-diameter bars were used for the stirrups. The bars featured surface ribs with a height of approximately 6% of their nominal diameter and were prepared with a length of 1980 mm. Representative images of the NCA, RCA, river sand, and BFRP bars are presented in Figure 1a–d, respectively. The particle size distributions of NCA and RCA are presented in Figure 2. The physical properties of the two coarse aggregates, including apparent density, water absorption, and crushing index, are summarized in Table 2. The mechanical properties of the BFRP bars, including tensile strength and elastic modulus, are summarized in Table 3, which are consistent with those reported in our previous study [34]. A polycarboxylate-based high-performance superplasticizer produced by Shanghai Xin Materials Co., Ltd. (Shanghai, China) was used to improve the workability of the concrete mixtures.

### 2.2. Test Methods

Earlier research, together with preliminary mixture trials, demonstrated that combining steel and PVA fibers can simultaneously improve the strength and crack resistance of RAC through their complementary effects [35,36]. Steel fibers, owing to their relatively high stiffness and tensile strength, are primarily effective in bridging and transferring stresses across macrocracks, whereas PVA fibers, with their smaller diameter and higher flexibility, contribute to the control of microcrack initiation and propagation and promote a more distributed cracking pattern. Therefore, the combined use of the two fiber types was considered beneficial for improving both the mechanical resistance and cracking characteristics of RAC. Preliminary trials were conducted to determine an appropriate steel fiber-to-PVA fiber volume ratio. As summarized in Table 4, the 1:1 ratio exhibited the highest 28-day cube compressive strength among the investigated combinations at both total fiber volume fractions of 0.6% and 1.2%. Therefore, a steel fiber-to-PVA fiber volume ratio of 1:1 was selected and kept constant throughout the experimental program. The total hybrid fiber (HF) content was varied from 0.6% to 1.5% by volume. Increasing the HF content to 1.5% resulted in reduced workability and may have hindered the uniform dispersion of fibers, which could contribute to fiber agglomeration and the subsequent reduction in mechanical performance. Based on these considerations, four total HF contents of 0.6%, 0.9%, 1.2%, and 1.5% were selected for the present study, with fiber-free RAC serving as the reference mixture.

The experimental program consisted of two stages. The first stage evaluated how the hybrid fiber (HF) dosage affected the fundamental mechanical properties of RAC. The second stage examined the flexural performance of BFRP-reinforced hybrid fiber-recycled aggregate concrete (HFRAC) beams, with particular emphasis on the effects of HF dosage and BFRP reinforcement ratio.

In the first stage, steel and PVA fibers were blended at a fixed volumetric ratio of 1:1. Four total HF volume fractions (0.6%, 0.9%, 1.2%, and 1.5%) were considered, together with a fiber-free RAC mixture (0%) and a conventional Portland cement concrete (PC) mixture with natural aggregate as reference mixtures. The six mixtures were designated as PC, RAC, HF06, HF09, HF12, and HF15, respectively. Their compressive and splitting tensile strengths as well as elastic modulus were evaluated, and the corresponding mix proportions are summarized in Table 5. Note that the 1:1 steel fiber-to-PVA fiber ratio represents a volume ratio, and the corresponding mass contents of the two fibers were determined according to their respective densities.

The second experimental stage comprised nine beam specimens designed to examine the influence of HF dosage and BFRP reinforcement ratio on the flexural performance of BFRP-reinforced RAC/HFRAC members. Each beam measured 120 mm in width, 200 mm in height, and 2000 mm in length, with a nominal concrete cover of 15 mm, which was selected in accordance with the requirements for Class I environmental exposure. BFRP bars were employed as the longitudinal reinforcement, while 8-mm-diameter BFRP stirrups were installed at 100 mm intervals in the shear spans outside the pure bending region. No stirrups were provided within the pure bending region to avoid interference with the development and observation of flexural cracks. The fiber-free RAC beam and the PC beam were used as reference specimens. For the BFRP-reinforced HFRAC beams, the HF content was varied from 0.6% to 1.5%, while the BFRP longitudinal reinforcement ratio was varied by changing the diameter of the longitudinal BFRP bars. To investigate the effect of the BFRP reinforcement ratio, the HF content was fixed at 1.2%, which showed the best performance among the investigated HF contents, while the diameter of the longitudinal BFRP bars was varied. Conversely, to investigate the effect of HF content, the BFRP reinforcement ratio was fixed at 1.10%, while the HF content was varied. Table 6 presents the key parameters of the beam specimens.

In the specimen designation, “B” indicates the use of BFRP bars as the lower longitudinal reinforcement, and the number following “B” represents the nominal diameter of the BFRP bars in millimeters. For example, R06-B12 denotes a recycled aggregate concrete beam incorporating 0.6% HF and reinforced with 12-mm-diameter BFRP bars as the lower longitudinal reinforcement. In the reinforcement notation, “2Φ8” denotes two erection bars with a nominal diameter of 8 mm, whereas “Φ8@100” represents 8-mm-diameter BFRP stirrups spaced at 100 mm.

**Figure 3:**
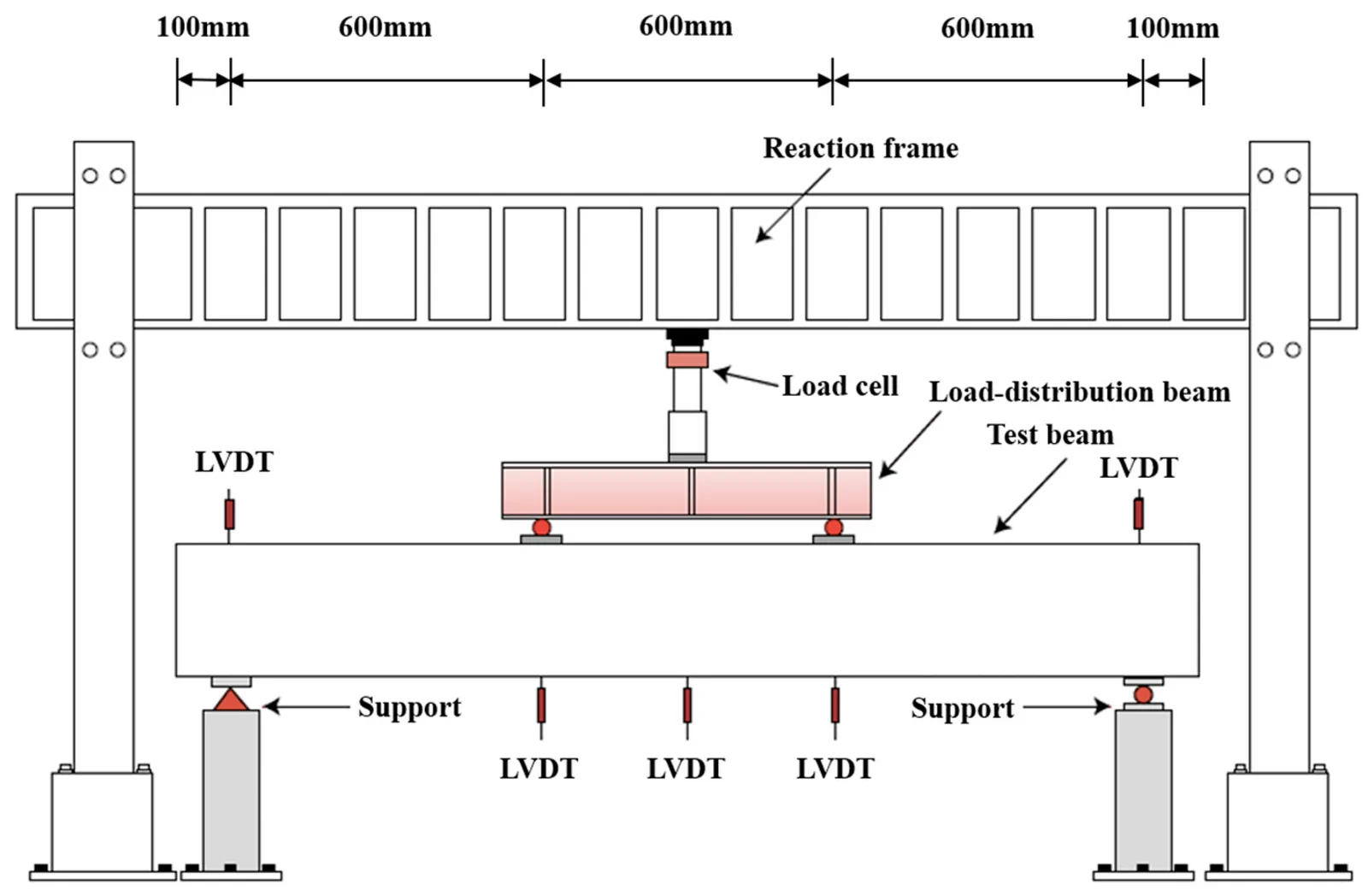
Configuration of the four-point bending test.

The basic mechanical performance of the RAC mixtures was assessed in terms of cube compressive strength, axial compressive strength, elastic modulus and splitting tensile strength. All tests were conducted using a 1000 kN electro-hydraulic servo universal testing machine, with three replicate specimens prepared for each mixture and test type. Cube compression, elastic modulus and splitting tensile tests employed 100 mm × 100 mm × 100 mm specimens, whereas axial compression was evaluated using 100 mm × 100 mm × 300 mm prisms. The elastic modulus was determined from the stress–strain response of the specimens under uniaxial compression, with axial deformation recorded by a displacement measurement system. The compression tests were carried out under displacement-controlled continuous loading at a constant rate of 8 kN/s. For the splitting tensile tests, a specialized loading fixture was used to apply the load to the cubic specimens. The flexural tests were performed under monotonic four-point loading with a 1000 kN-capacity electro-hydraulic universal testing machine. The experimental setup and loading configuration are schematically illustrated in Figure 3. Before formal loading, a preloading procedure was conducted to eliminate potential gaps between the loading system and the beam specimens and to ensure uniform contact between the loading points and the beam surface. During preloading, the load was applied incrementally at a displacement rate of 0.2 mm/min, with a load increment of 2 kN and a holding period of 3 min at each load level. The specimens were loaded to 4 kN and then unloaded, and this procedure was repeated twice. If any gaps were observed between the loading points and the beam surface, fine sand was inserted to improve contact uniformity.

The formal loading was performed under displacement control. Before the initiation of the first crack, the load was increased in increments of 2 kN at a displacement rate of 0.2 mm/min, with each load level maintained for 3 min. After the first flexural crack was observed, the load increment was increased to 5 kN and the displacement rate was increased to 0.5 mm/min, while the holding period remained unchanged. The test was terminated when either tensile fracture of the BFRP longitudinal bars or crushing of the concrete matrix in the compression zone occurred. These failure events generally corresponded to the attainment of the peak load followed by a descending branch in the load–displacement response. Five LVDTs were positioned at selected points along the beams to capture their vertical displacement response. A load cell installed beneath the hydraulic actuator was used to continuously record the applied load. The crack widths were measured using a digital crack microscope with an accuracy of 0.01 mm. Measurements were conducted at the maximum crack opening region within the pure bending zone, and the largest measured crack width before failure was defined as the maximum crack width.

## 3. Experimental Results and Analysis

### 3.1. Basic Mechanical Properties

Table 7 summarizes the 28-day compressive and splitting tensile strength as well as elastic modulus results obtained for the concrete mixtures. The values in parentheses represent the standard deviations obtained from three specimens. Table 7 indicates that increasing the HF content initially enhanced the cube compressive strength of HFRAC, whereas further fiber addition beyond 1.2% led to a reduction in strength. Compared with the fiber-free RAC, the cube compressive strengths of HF06, HF09, HF12, and HF15 increased by 1.45%, 6.18%, 19.44%, and 2.45%, respectively. The elastic modulus of HF06, HF09, HF12, and HF15 increased by 1.78%, 4.27%, 11.39%, and 2.14%, respectively. This trend is consistent with those reported in previous studies [35], in which the compressive strength of fiber-reinforced concrete generally increases initially and then decreases with increasing fiber content, with the optimum fiber content typically depending on the concrete matrix and fiber system.

The enhanced mechanical properties are mainly associated with the crack-bridging action provided by the hybrid fiber system. At an appropriate fiber content, the steel and PVA fibers bridge developing cracks and restrict crack propagation within the concrete matrix, thereby improving its resistance to damage [36]. In addition, the abundant hydrophilic hydroxyl groups (-OH) on the surface of PVA fibers enable them to absorb and retain water, which may promote the hydration of the cementitious matrix and contribute to strength development. However, when the HF content is excessive, fiber agglomeration may occur within the matrix, potentially resulting in nonuniform fiber dispersion and increased internal defects. This weakens the reinforcing effect of the fibers. Moreover, excessive PVA fibers can absorb a considerable amount of mixing water during concrete mixing, reducing the workability and fluidity of the fresh mixture and consequently limiting the development of compressive strength.

The axial compressive and splitting tensile strength results exhibited a consistent pattern. Compared with the fiber-free RAC, the axial compressive strengths of HF06, HF09, HF12, and HF15 increased by 3.11%, 8.07%, 23.08%, and 4.43%, respectively, while the corresponding increases in splitting tensile strength were 5.51%, 22.05%, 32.55%, and 18.37%, respectively. The enhancement was particularly pronounced when the HF content increased from 0.6% to 1.2%, with the maximum improvements of 23.08% in axial compressive strength and 32.55% in splitting tensile strength achieved at 1.2% HF. When the HF content was further increased to 1.5%, the enhancement effect decreased substantially. These results indicate that, among the investigated HF contents, 1.2% provided the best overall basic mechanical performance, which may be attributed to a favorable balance between fiber bridging, crack restraint, and fiber dispersion.

It is also noteworthy that the mechanical properties of HF12 exceeded those of PC, indicating that the incorporation of an appropriate amount of HFs can not only compensate for the mechanical deterioration caused by recycled aggregates but also enable HFRAC to achieve mechanical performance superior to that of conventional PC.

The axial-to-cube compressive strength ratio decreased from 0.823 for PC to 0.797 for RAC, indicating that the use of recycled aggregate slightly reduced the axial compressive strength relative to the cube compressive strength. With increasing HF content, this ratio increased to 0.821 at 1.2% HF, approaching the value of PC, before slightly decreasing to 0.812 at 1.5% HF. In contrast, the splitting tensile-to-cube compressive strength ratio increased from 0.093 for RAC to 0.104 at 1.2% HF, demonstrating that the incorporation of hybrid fibers produced a more pronounced improvement in tensile performance than in compressive strength. The subsequent decrease to 0.103 at 1.5% HF further confirms that excessive fiber content weakened the overall reinforcing efficiency.

### 3.2. Flexural Behavior of Beams

#### 3.2.1. Typical Failure Mode

Three characteristic failure modes were identified from the failure development and damage patterns observed in the nine beam specimens:

(1) Crushing failure of concrete in the compression zone (over-reinforcement failure)

Specimens R12-B14 and R12-B16 exhibited this failure mode, corresponding to reinforcement ratios of 1.51% and 1.98%, respectively. The failure process is characterized by the following features: concrete crushing first developed in the compression zone, followed by localized arching and spalling of the upper concrete layer; the BFRP bars remain intact, and the deformed portions recover after unloading; cracks are numerous, fine, and exhibit a branching pattern; the overall failure process is relatively gradual and mild.

**Figure 4:**
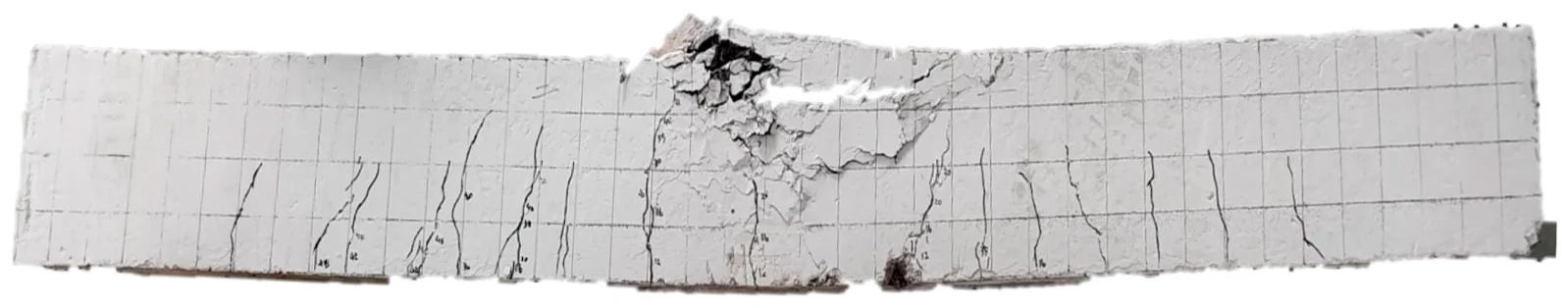
Failure mode observed in specimen R12-B14.

At approximately 80% of the ultimate load, the onset of horizontal cracking was observed within the compression zone. Subsequently, the concrete gradually bulged, ultimately forming a continuous failure surface. Since the BFRP bars did not fracture, the specimen retained a certain residual load-carrying capacity. Although the strength of the BFRP bars was not fully utilized, clear signs of failure were evident. Figure 4 shows a physical image of the failure mode of specimen R12-B14.

(2) Tensile failure of the BFRP bar (under-reinforcement failure)

Specimen R12-B8 exhibited this failure pattern at a reinforcement ratio of 0.48%. The failure initiates with sudden fracture of the BFRP bars, accompanied by audible cracking. The compression-zone concrete remained generally undamaged, with only limited evidence of crushing. Cracks are primarily concentrated in the pure bending and bending-shear regions, with a small number of main cracks but relatively large widths. The failure exhibits a brittle nature, and the residual deformations are irreversible.

**Figure 5:**
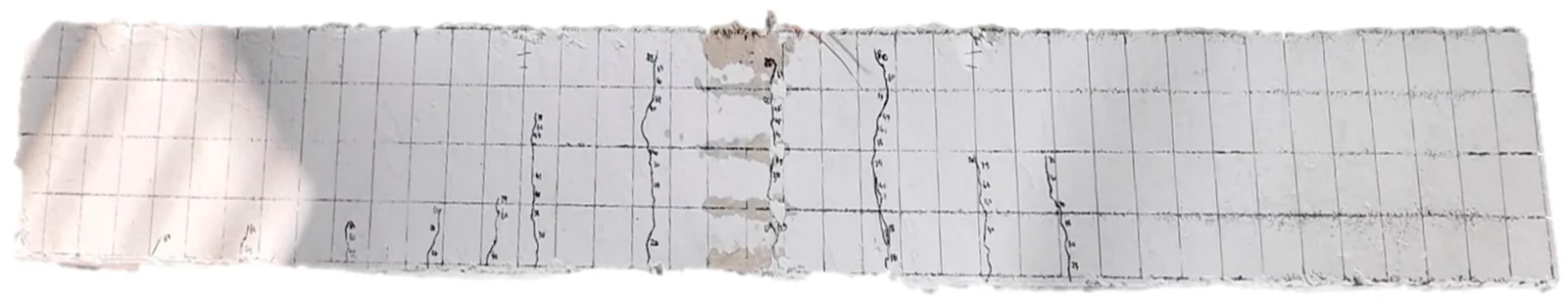
Tensile rupture of the BFRP bars in specimen R12-B8.

Under low reinforcement ratios, the tensile stress carried by the BFRP bars constitutes a large proportion of the applied stress, and stress develops rapidly. BFRP bars are linear elastic materials without a yield plateau and fail in a brittle manner once the tensile stress reaches their ultimate strength. In this failure mode, the strain of the concrete in the compression zone does not reach the ultimate compressive strain, and the material’s strength potential is not fully utilized. Such failures occur suddenly, with few observable precursors, and should be avoided in design. Figure 5 shows a physical image of the failure mode of specimen R12-B8.

(3) Equilibrium failure (balanced failure)

This failure mode was observed in specimens R12-B12, R06-B12, R09-B12, R15-B12 (with a reinforcement ratio of 1.10%) and the control groups PC-B12 and RAC-B12. The tensile failure of the BFRP bars and the crushing of concrete in the compression zone occur almost simultaneously. Prior to failure, the specimens exhibit obvious deformations and fully developed cracks. This mode combines ductile behavior with efficient material utilization, representing a balanced interaction between tensile and compressive elements.

**Figure 6:**
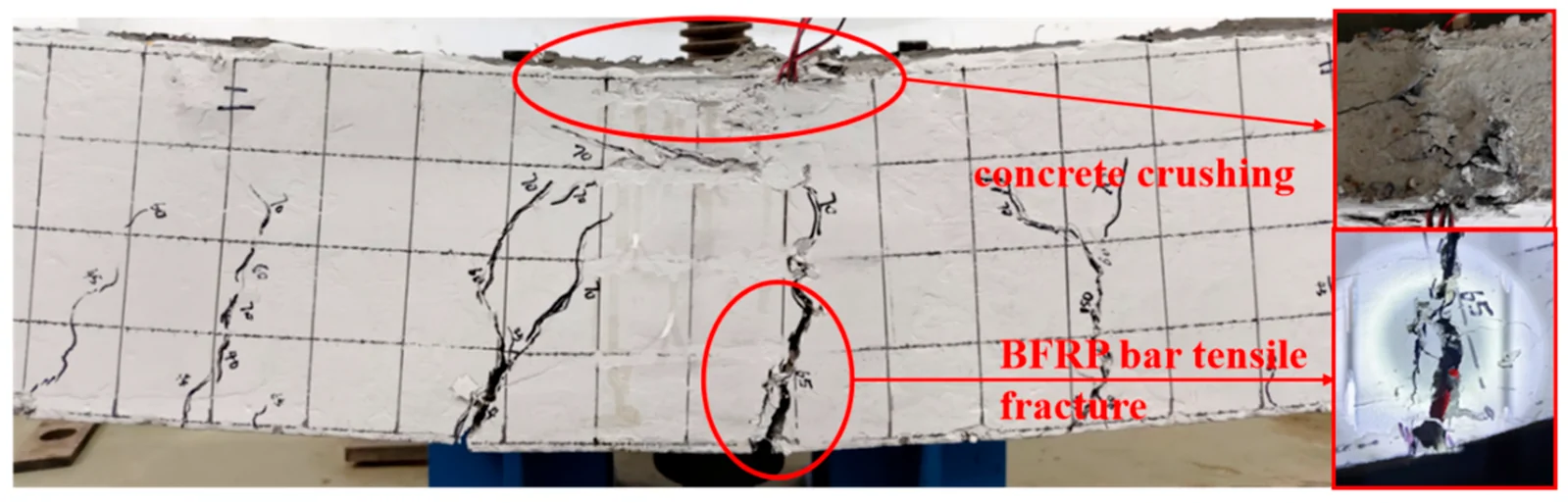
Balanced failure of the beam in specimen R12-B12.

This failure mode resembles the ultimate failure of conventional reinforced concrete beams and represents an ideal design objective. In the present study, a reinforcement ratio of 1.10% corresponds to the critical point of balanced failure, at which both the ultimate compressive strain of concrete and the ultimate tensile strain of the BFRP bars are simultaneously achieved. Figure 6 shows the physical appearance of the failure mode of specimen R12-B12.

The role of HF dosage in governing the failure pattern was assessed while maintaining the BFRP reinforcement ratio at 1.10%. Irrespective of the fiber dosage, ranging from 0 to 1.5%, all five beam specimens developed a balanced failure mode, indicating that HF addition did not alter the fundamental failure classification. Nevertheless, the presence of hybrid fibers noticeably enhanced the deformation capacity and ductile response of the beams during the failure process. In the non-fiber specimen RAC-B12, cracks developed rapidly and failure occurred suddenly, whereas in R12-B12, cracks developed gradually. The bridging effect of the fibers refined the crack pattern and distributed cracks more uniformly, providing observable precursors prior to failure. Compared with RAC-B12, R12-B12 showed an 84.2% increase in initial cracking load and a 47.9% reduction in maximum crack width at failure, while exhibiting similar mid-span deflection but substantially improved crack control. These results demonstrate that hybrid fibers enhance the performance of BFRP-reinforced beams by inhibiting crack development, without altering the classification of failure modes.

The reinforcement ratio was found to be a key factor in determining the type of failure. In this study, beams with reinforcement ratios below 0.9% exhibited BFRP tensile failure, ratios between 1.0% and 1.1% led to balanced failure, and ratios above 1.5% resulted in concrete crushing. With a fiber content of 1.2%, these boundaries are generally consistent with the design provisions for FRP-reinforced beams in GB 50608-2020 [37], indicating that hybrid fibers do not significantly alter the criteria for determining failure modes. Notably, the presence of fibers slightly broadens the ductile response range. For example, R12-B12 (ρ = 1.10%) exhibits ideal balanced failure characteristics, whereas RAC-B12, without fibers but with the same reinforcement ratio, tends toward over-reinforcement characteristics due to the brittleness of concrete, with concrete crushing occurring slightly before the fracture of the BFRP bars. This highlights that the incorporation of fibers improves concrete ductility and enhances the synergistic interaction between BFRP bars and concrete.

#### 3.2.2. Load-Deflection Behavior

(1) Load-deflection response of the test beams

**Figure 7:**
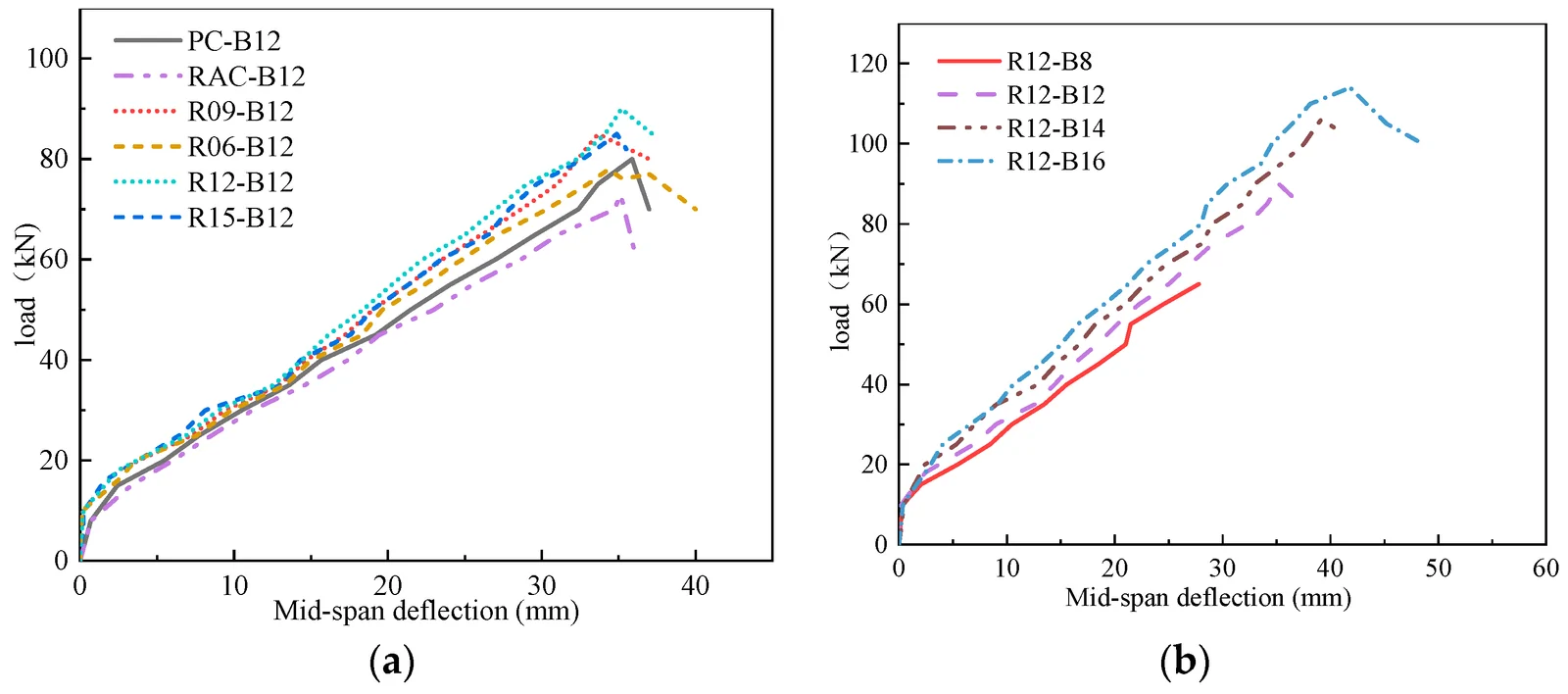
Flexural load-midspan deflection response of the test beams. (**a**) Different fiber content. (**b**) Different reinforcement ratios.

The load-deflection curve of BFRP-reinforced concrete beams exhibits a double-fold line shape, which is significantly different from the curve shape of reinforced concrete beams. The load-midspan deflection curves of each group of specimens are shown in Figure 7, which can be divided into three stages:

The first stage occurs before cracks appear in the beam span, where the curve exhibits linear growth with a large slope. In this stage, the concrete is not cracked, and the BFRP rebar works in concert with the concrete. The section stiffness is primarily controlled by the elastic modulus of the concrete. Since the elastic modulus of BFRP rebar (approximately 41 GPa) is only 1/5 of that of steel rebar, but the reinforcement ratio is relatively high, the stiffness in this stage is slightly lower than that of reinforced concrete beams of the same size.

The second stage occurs after cracks appear in the beam span. After the cracks appear, the slope of the curve suddenly decreases, but it still maintains an approximate linear growth. This is because the concrete no longer participates in tension after cracking, and the tensile force is mainly borne by the BFRP bars. The linear elastic properties of the BFRP bars make this stage lack a yield platform, exhibiting a “double straight line” characteristic. The slope in this stage directly reflects the level of BFRP bar reinforcement ratio, that is, the higher the reinforcement ratio, the greater the slope, and the smaller the stiffness degradation.

The third stage occurs after the load reaches the ultimate load of the test specimen, during which the load-deflection curve exhibits a sharp decline. The BFRP rebar breaks or the concrete is crushed, resulting in a sudden loss of the component’s bearing capacity. This is manifested as a continuous increase in deflection and a rapid decrease in load, exhibiting significant brittle failure characteristics.

Figure 7 depicts the load-mid-span deflection curve of the component. When the hybrid fiber (HF) content ranges from 0.6% to 1.2%, the mid-span deflection of the beam decreases with increasing content, indicating that an appropriate amount of fiber can effectively inhibit crack propagation and enhance the stiffness of the component. However, when the HF content increased to 1.5%, the stiffness decreased, which may be associated with reduced workability and difficulties in achieving uniform fiber dispersion at a higher fiber dosage, potentially affecting the continuity of the concrete matrix. Additionally, the reinforcement ratio has a significant impact on deflection development: increasing the reinforcement ratio can increase the ultimate deflection but effectively reduce deformation under the same load level. For example, when the load is 60 kN and the reinforcement ratio increases from 0.48% to 1.98%, the mid-span deflection decreases by 22.29%, indicating that a high reinforcement ratio significantly enhances the stiffness of the section after cracking.

(2) Initial cracking load and ultimate load of the test beams

**Figure 8:**
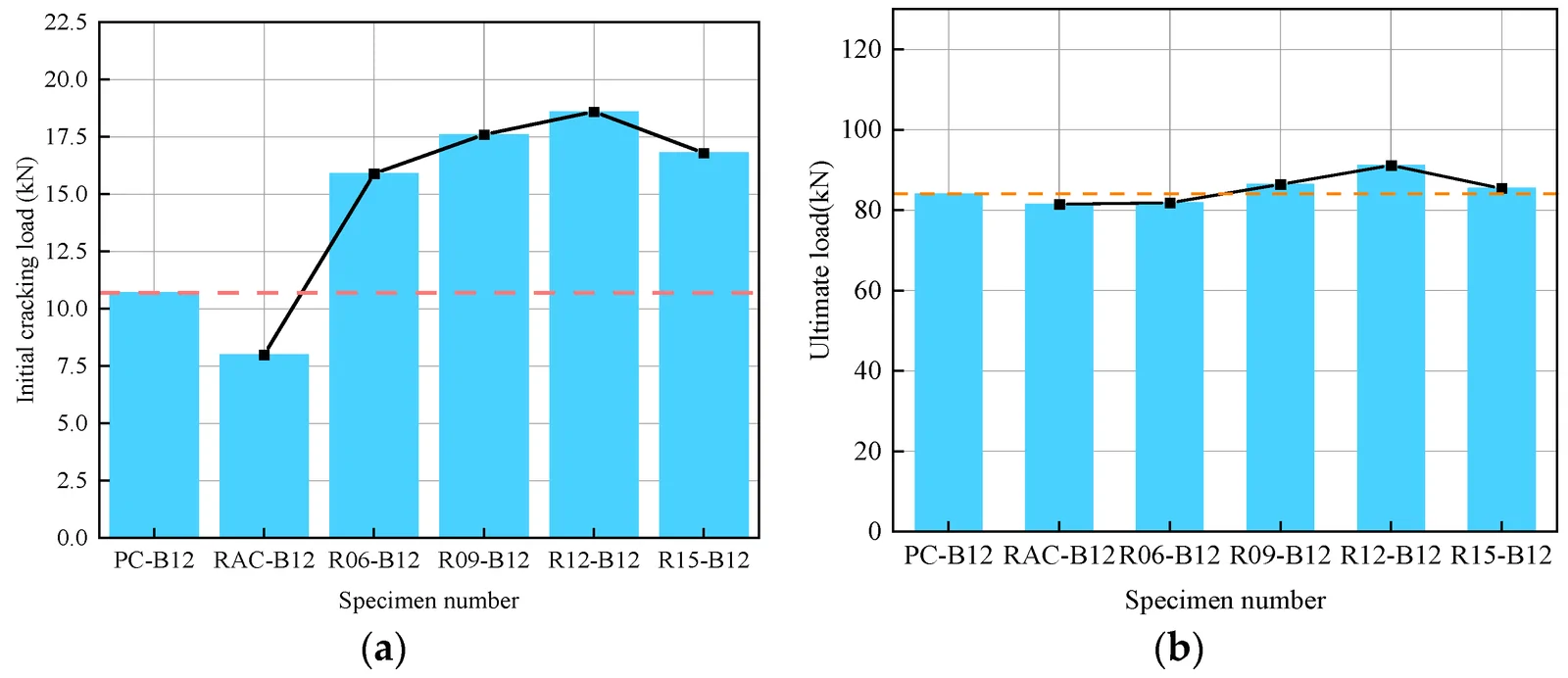
Comparison of test beams with different HF dosages. (**a**) Initial cracking load. (**b**) Ultimate load.

The influence of hybrid fiber content was investigated for beams with the same BFRP reinforcement ratio within an appropriate reinforcement range. As shown in Figure 8, both the initial cracking load and ultimate load of hybrid fiber-reinforced recycled concrete (HFRAC) beams exhibit a nonlinear trend with increasing fiber content, rising initially and then decreasing. Compared to recycled concrete beams without fibers (RAC), the initial cracking load increases by 98.75%, 120.14%, 132.51%, and 110.04% for fiber contents of 0.6%, 0.9%, 1.2%, and 1.5%, respectively, while the ultimate load increases by 0.47%, 6.09%, 11.92%, and 4.87%, respectively. These results indicate that hybrid fibers have a more pronounced effect on crack resistance than on ultimate bearing capacity.

Further comparison with ordinary concrete (PC) beams reveals that the initial cracking load and ultimate load of RAC beams are lower than those of PC beams. The incorporation of an appropriate amount of hybrid fibers effectively compensates for the performance deficiencies of recycled aggregates. Specifically, for fiber contents of 0.6%, 0.9%, 1.2%, and 1.5%, the initial cracking load of HFRAC beams increases by 48.60%, 64.49%, 73.83%, and 57.01%, respectively, compared to PC beams without fibers. The ultimate load increases by 2.78%, 8.43%, and 1.61% for fiber contents of 0.9%, 1.2%, and 1.5%, respectively. These findings demonstrate that the introduction of PVA/steel hybrid fibers not only mitigates the adverse effects of inherent defects in recycled aggregates, but also enhances the crack resistance and load-bearing capacity of HFRAC beams beyond those of ordinary concrete beams, providing an effective strategy for high-performance applications of recycled concrete in structural engineering.

**Figure 9:**
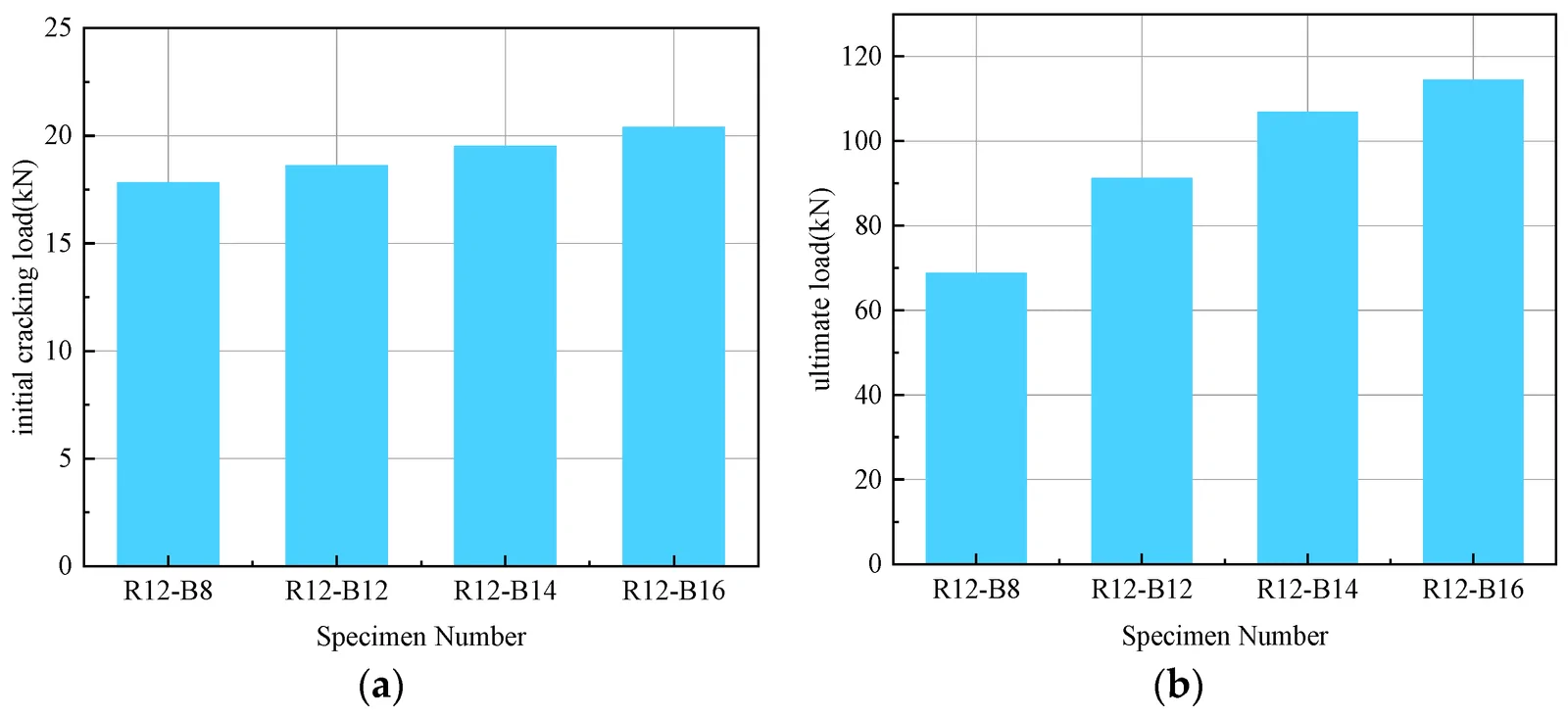
Comparison of test beams with different BFRP reinforcement ratios. (**a**) Initial cracking load. (**b**) Ultimate load.

The influence of the BFRP reinforcement ratio was also evaluated under a constant HF content. Figure 9 demonstrates that increasing the reinforcement ratio resulted in higher initial cracking and ultimate loads. However, their sensitivities differ significantly. The reinforcement ratio has a limited effect on the initial cracking load: compared to the baseline ratio of 0.48%, increasing the ratio to 1.10%, 1.51%, and 1.98% results in initial cracking load increases of 4.49%, 9.55%, and 14.61%, respectively. This indicates that the cracking moment of the section is primarily controlled by the tensile strength of the concrete matrix, with the contribution of the reinforcement being relatively small. In contrast, the effect of the reinforcement ratio on the ultimate load is substantial: under the same increase in reinforcement ratio, the ultimate load rises by 32.60%, 55.35%, and 66.37%, showing an approximately linear growth trend. These patterns reflect the essential mechanical behavior of reinforced beams: the reinforcement ratio governs the ultimate-state performance by altering the internal force lever arm and the tensile capacity of the FRP bars in the tension zone, while exerting a weaker influence on cracking behavior in the elastic stage.

#### 3.2.3. Crack Propagation and Development

(1) Crack patterns and distribution

**Figure 10:**
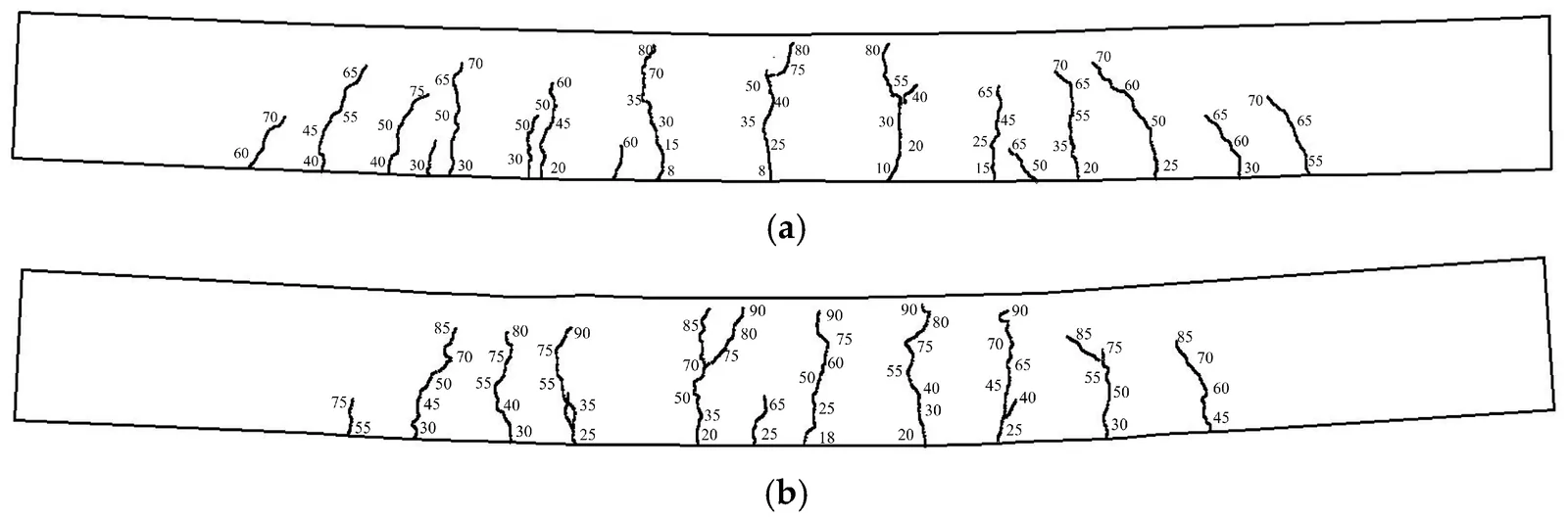
Cracking patterns of representative test beams. (**a**) RAC-B12 beam. (**b**) R12-B12 beam.

Figure 10 presents the crack distribution patterns of representative specimens. The fiber-free beam RAC-B12 exhibited numerous visible cracks with irregular spacing and a scattered distribution of major cracks. In contrast, the hybrid fiber-reinforced beam R12-B12 showed fewer visible macrocracks, with cracks mainly concentrated in the mid-span region and exhibiting a more uniform distribution. Although the number of visible cracks was reduced, the crack widths were significantly decreased, indicating an effective crack-width control effect of the hybrid fiber system. This phenomenon can be attributed to the combined crack-control mechanisms provided by hybrid fibers and BFRP reinforcement. The bridging effect of steel fibers restricted the opening and propagation of macrocracks, while PVA fibers improved the tensile resistance of the cementitious matrix and delayed microcrack development. Consequently, tensile stresses were redistributed more gradually, preventing the rapid localization of damage into a few wide cracks. It should be noted that fiber reinforcement does not always result in a higher number of visible cracks. For strain-softening fiber-reinforced concrete systems, fibers mainly contribute to controlling crack opening and stabilizing crack propagation rather than inducing extensive multiple cracking as observed in strain-hardening cementitious composites. Similar observations were reported by Ghahremannejad et al., who found that the incorporation of synthetic fibers in reinforced concrete beams reduced both the number and width of visible cracks due to the crack-bridging effect of fibers [38]. In addition, the improved tensile resistance of the fiber-reinforced matrix and the restraining effect of BFRP reinforcement reduced tensile strain concentration under flexural loading, leading to fewer but narrower visible cracks in the R12-B12 beam compared with the fiber-free RAC-B12 beam.

(2) Crack width control

**Figure 11:**
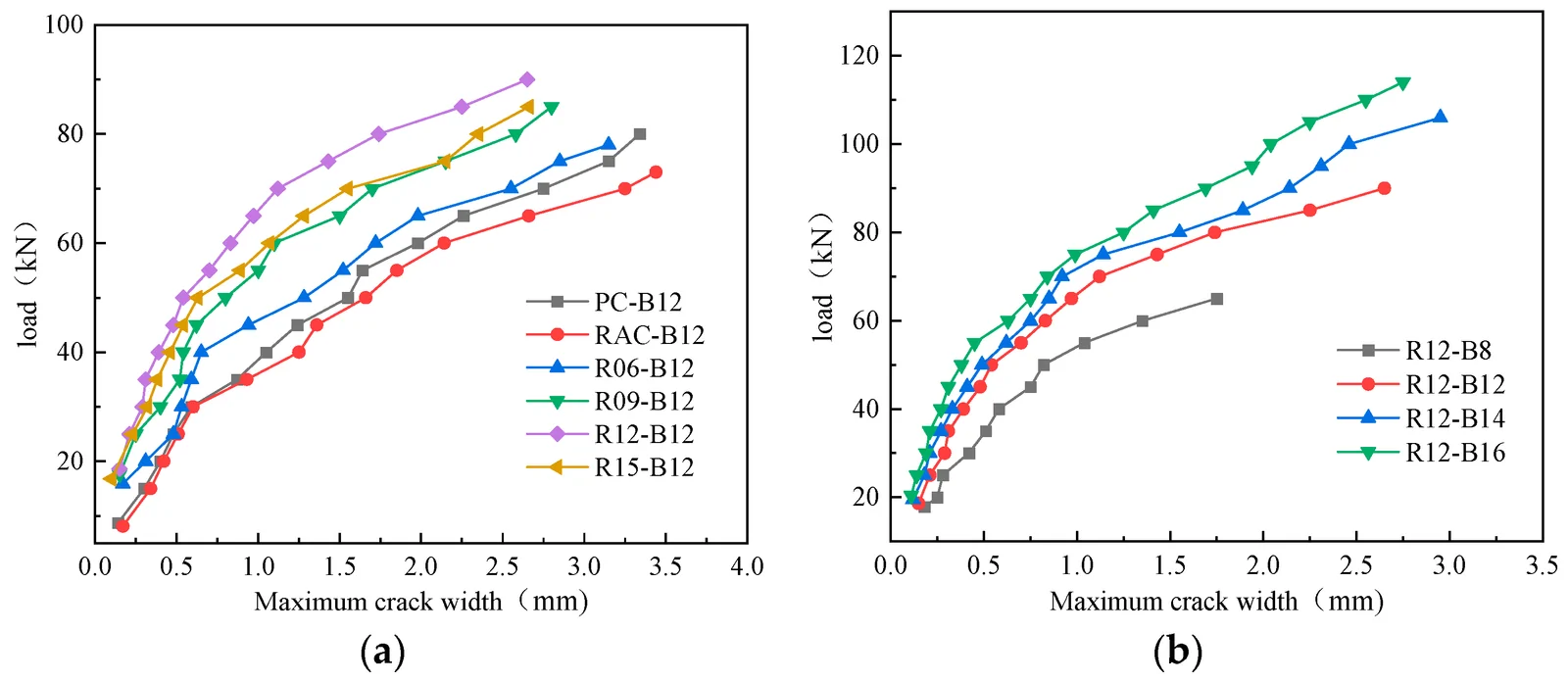
Variation in maximum crack width with HF dosage and BFRP reinforcement ratio. (**a**) HF dosage. (**b**) BFRP reinforcement ratio.

Figure 11a illustrates the effect of hybrid fiber content on the crack width of beams. The curves indicate a clear correlation between fiber content and crack control performance, with an apparent threshold effect. The specimen R12-B12, with 1.2% hybrid fiber content, exhibits the smallest crack width of 1.17 mm under a load of 65 kN, representing a 56% reduction compared to fiber-free RAC beams and a significant improvement over ordinary concrete (PC) beams. The R09-B12 specimen shows slightly larger cracks but follows a similar trend, whereas R06-B12, with insufficient fiber content, demonstrates limited effectiveness, closely resembling PC and RAC beams. In contrast, the R15-B12 specimen exhibits wider cracks than R12-B12, suggesting that the higher fiber content may adversely affect crack resistance, possibly due to reduced workability and difficulties in achieving uniform fiber dispersion. Among the investigated fiber contents, 1.2% HF exhibited the most favorable crack-control performance. Moreover, the slope of the R12-B12 curve is the lowest, indicating that fibers slow the crack propagation rate through bridging action and promote uniform and controlled crack development.

Figure 11b presents the influence of BFRP reinforcement ratio on crack width. The curves are arranged in descending order of reinforcement ratio, with R12-B16 exhibiting the smallest crack width at approximately 0.7 mm under a load of 60 kN, which is 65% lower than that of R12-B8. This demonstrates that increasing the reinforcement ratio can significantly limit crack development. However, the mechanisms differ from those of fiber addition: a high reinforcement ratio reduces crack width by lowering the stress level in the BFRP bars, but also increases beam stiffness and reduces ductility. The increased slope of the R12-B16 curve in the later stage reflects sudden crack development prior to concrete crushing, whereas the R12-B8 specimen, with a lower reinforcement ratio, shows larger cracks but a smaller slope, indicating greater deformation capacity and more pronounced precursors before failure. It should be noted that, unlike steel-reinforced concrete beams, where crack spacing is often influenced by bar diameter and bar distribution, the crack behavior of BFRP-reinforced beams is strongly affected by the reinforcement ratio and failure mode. Increasing the BFRP reinforcement ratio enhances section stiffness and reduces tensile strain, resulting in smaller crack widths and spacing under comparable loading conditions [39].

Overall, comparison of the two sets of results indicates that 1.2% hybrid fibers are more effective for crack control than merely increasing the reinforcement ratio. Accordingly, an appropriate combination of hybrid fiber incorporation and BFRP reinforcement ratio (approximately 1.0–1.5%) can provide a favorable compromise between crack-width control and ductile structural response.

## 4. Conclusions

Based on the experimental results of the basic mechanical properties and four-point bending tests of nine beam specimens, the following conclusions can be drawn:

(1) The incorporation of hybrid fibers effectively mitigated the mechanical deterioration of RAC associated with recycled aggregates. The cube compressive strength, axial compressive strength, elastic modulus, and splitting tensile strength of HFRAC initially increased and subsequently decreased with increasing HF content, with 1.2% HF providing the best performance among the investigated fiber contents. Compared with fiber-free RAC, the 1.2% HF content increased the four mechanical parameters by 19.44%, 23.08%, 11.39%, and 32.55%, respectively. The results indicate that an appropriate HF content can effectively improve the mechanical performance of RAC under the investigated conditions.
(2) Three typical failure modes were identified in the beam tests, namely BFRP bar rupture, balanced failure, and concrete crushing. BFRP bar rupture occurred at relatively low reinforcement ratios, balanced failure was observed at reinforcement ratios of approximately 1.0–1.1%, and concrete crushing occurred at higher reinforcement ratios. The incorporation of hybrid fibers did not alter the fundamental failure-mode classification of the investigated beams.
(3) Hybrid fibers showed a more pronounced effect on crack control than on ultimate load capacity. Compared with the fiber-free RAC beam, the beam incorporating 1.2% HF exhibited increases of 132.51% and 11.92% in cracking and ultimate loads, respectively, while the crack width was reduced by 47.9%. Under the investigated conditions, the 1.2% HFRAC beam demonstrated improved flexural performance compared with the reference beams. Further increasing the HF content to 1.5% resulted in performance deterioration, which may be associated with reduced workability and difficulties in achieving uniform fiber dispersion at higher fiber dosages.
(4) The BFRP reinforcement ratio significantly influenced the ultimate load capacity, which showed an increasing trend with increasing reinforcement ratio, while its effect on cracking load was relatively limited. Increasing the reinforcement ratio enhanced the flexural stiffness but reduced the deformation capacity. Lower reinforcement ratios resulted in larger crack widths and a higher likelihood of sudden BFRP bar rupture.
(5) Hybrid fibers effectively improved crack control through their bridging effect and refined the crack distribution. The 1.2% HF content provided more effective crack control than increasing the BFRP reinforcement ratio alone within the investigated range. The load-deflection responses of the BFRP-reinforced beams exhibited bilinear characteristics without an apparent yield plateau, followed by a rapid reduction in load-carrying capacity after reaching the ultimate load.

It should be noted that each beam configuration was represented by a single specimen in the present experimental program, which limits the statistical validation of the observed trends in flexural behavior. Therefore, the findings of this study should be interpreted within the scope of the investigated parameters. Further experimental studies involving a larger number of specimens are required to statistically verify the effects of hybrid fiber content and BFRP reinforcement ratio and to establish more generalized design recommendations.

## Figures and Tables

**Table 1:** Properties of Steel and Polyvinyl Alcohol (PVA) Fibers.

Fiber Type	Morphology	Diameter/μm	Length/mm	Density/(kg/m^3^)	Elongation at Break/%	Tensile Strength/MPa
Steel fiber	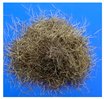	300	12	7800	1.8	1150
PVA fiber	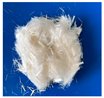	30	12	1210	19.6	760

**Table 2:** Physical properties of NCA and RCA.

Aggregate Type	Apparent Density (kg/m^3^)	Crushing Index (%)	Water Absorption%
NCA	2760	7.45	0.86
RCA	2340	18.12	5.71

**Table 3:** Mechanical characteristics of BFRP bars [34].

Diameter (mm)	Tensile Strength (MPa)	Modulus of Elasticity (GPa)
8	921	44.3
12	980	41.9
14	1030	41
16	1069	40.5

**Table 4:** Preliminary evaluation of steel-to-PVA fiber volume ratio based on 28-day cube compressive strength.

Total Fiber Volume Fraction (%)	Steel:PVA Fiber Ratio	28-Day Compressive Strength (MPa)
0.6	1:0	38.5
0.6	2:1	40.6
0.6	1:1	41.4
0.6	1:2	39.7
0.6	0:1	36.2
1.2	1:0	43.8
1.2	2:1	47.1
1.2	1:1	48.7
1.2	1:2	45.9
1.2	0:1	41.6

**Table 5:** Concrete mixture proportions. (kg/m^3^).

Serial Number	Cement	Water	Fly Ash	River Sand	Recycled Aggregate	Natural Aggregate	Superplasticizer	Steel Fiber Content	PVA Fiber Content
PC	477	200	100	540	0	1280	6	0	0
RAC	477	200	100	540	1280	0	6	0	0
HF06	477	200	100	540	1280	0	6	23.64	3.66
HF09	477	200	100	540	1280	0	6	35.41	5.49
HF1.2	477	200	100	540	1280	0	6	47.27	7.33
HF1.5	477	200	100	540	1280	0	6	59.04	9.16

**Table 6:** Key parameters of the test beams.

Serial Number	Erection Bar	Stirrup	Longitudinal Reinforcement	Reinforcement Ratio	HF Content
PC-B12	2Φ8	Φ8@100	2B12	1.10%	0
RAC-B12			2B12	1.10%	0
R12-B12			2B12	1.10%	1.2%
R12-B8			2B8	0.48%	1.2%
R12-B14			2B14	1.51%	1.2%
R12-B16			2B16	1.98%	1.2%
R06-B12			2B12	1.10%	0.6%
R09-B12			2B12	1.10%	0.9%
R15-B12			2B12	1.10%	1.5%

**Table 7:** 28-day basic mechanical properties of PC, RAC, and HFRAC.

Specimen	Cube Compressive Strength (MPa)	Axial Compressive Strength (MPa)	Elastic Modulus (GPa)	Splitting Tensile Strength (MPa)	Axial-to-Cube Compressive Strength Ratio	Splitting Tensile-to-Cube Compressive Strength Ratio
PC	47.3 (1.6)	38.9 (1.5)	30.0	3.86 (0.7)	0.823	0.081
RAC	40.8 (2.3)	32.5 (2.6)	28.1	3.81 (1.0)	0.797	0.093
HF06	41.4 (1.6)	33.5 (3.1)	28.6	4.02 (0.8)	0.810	0.101
HF09	43.3 (2.2)	35.1 (3.5)	29.3	4.65 (1.5)	0.811	0.103
HF1.2	48.7 (0.8)	40.0 (2.4)	31.3	5.05 (2.0)	0.821	0.104
HF1.5	41.8 (1.9)	34.0 (0.7)	28.7	4.51 (1.7)	0.812	0.103

## Data Availability

The original contributions presented in this study are included in the article. Further inquiries can be directed to the corresponding author.
